# P-2309. Assessment of Risk Factors Associated with Subtherapeutic Posaconazole Levels

**DOI:** 10.1093/ofid/ofae631.2461

**Published:** 2025-01-29

**Authors:** Christopher Garzia, Brian Nelson, Angela Loo, Nicholas Demenagas, Christine J Kubin

**Affiliations:** NewYork-Presbyterian Hospital, New York, New York; NewYork-Presbyterian Hospital, New York, New York; New York-Presbyterian Hospital, New York, New York; NYP/Columbia University Irving Medical Center, New York, New York; NewYork-Presbyterian Hospital, New York, New York

## Abstract

**Background:**

There is significant variability in patient serum posaconazole levels, and it is unclear what characteristics increase the risk for subtherapeutic levels. Higher levels have been associated with improved outcomes. The purpose of this study is to identify risk factors associated with subtherapeutic posaconazole serum levels.

**Methods:**

Retrospective study of adult patients admitted to NewYork-Presbyterian Hospital who received posaconazole with at least one level obtained at steady-state. Levels were obtained after at least five consecutive days of therapy with no interruptions > 24 hours or dosage changes prior to initial level. Patients were grouped based on if the initial level was therapeutic, defined as a serum level > 1 mcg/mL. Risk factor assessment was performed using a logistic regression model. Baseline characteristics between therapeutic and subtherapeutic groups were compared via chi-squared test for categorical variables and independent t-test for continuous variables.

**Results:**

383 patients were included: 280 initial levels were therapeutic and 103 were subtherapeutic. Patients primarily consisted of those with solid organ transplant (48.8%) and hematologic malignancy (38.6%). Significant differences between therapeutic and subtherapeutic groups included median weight (73 vs 78 kg, p=0.017) and initial weight-based doses (4.4 vs 4.2 mg/kg, p=0.044), respectively. This prompted an exploratory analysis finding significant association with BMI > 35 (4.1% therapeutic vs 12.1% subtherapeutic, p=0.006). Initial dose thresholds of at least 3 mg/kg and 4 mg/kg showed nonsignificant increases in therapeutic levels (p=0.056 and p=0.107 respectively). On multivariable analysis, subtherapeutic levels were more likely in patients who are black or African American (OR 1.869, 95% CI 1.009, 3.465), had diarrhea (OR 1.978, 95% CI 1.149, 3.408), and had a BMI > 35 (OR 3.538, 95% CI 1.300, 9.628).

**Conclusion:**

Patients who are Black or African American, have diarrhea, and have a BMI > 35 were identified as having increased risk for subtherapeutic levels at 300 mg daily dosing. Patients at risk may benefit from initial weight-based dosing of at least 4mg/kg, however this requires additional confirmatory studies.

**Disclosures:**

All Authors: No reported disclosures

